# Supplementary material for: Novel Driver Strength Index highlights important cancer genes in TCGA PanCanAtlas patients
Source: PeerJ. 2022 Aug 11;10:e13860. doi: 10.7717/peerj.13860 (PMC9375969; doi:10.7717/peerj.13860)
Supplement: Supplemental Information 2 [file peerj-10-13860-s002.zip › COHORTS/genes plots/detailed/2021_8_16_14_9_distribution_events_detailed_43.pdf]

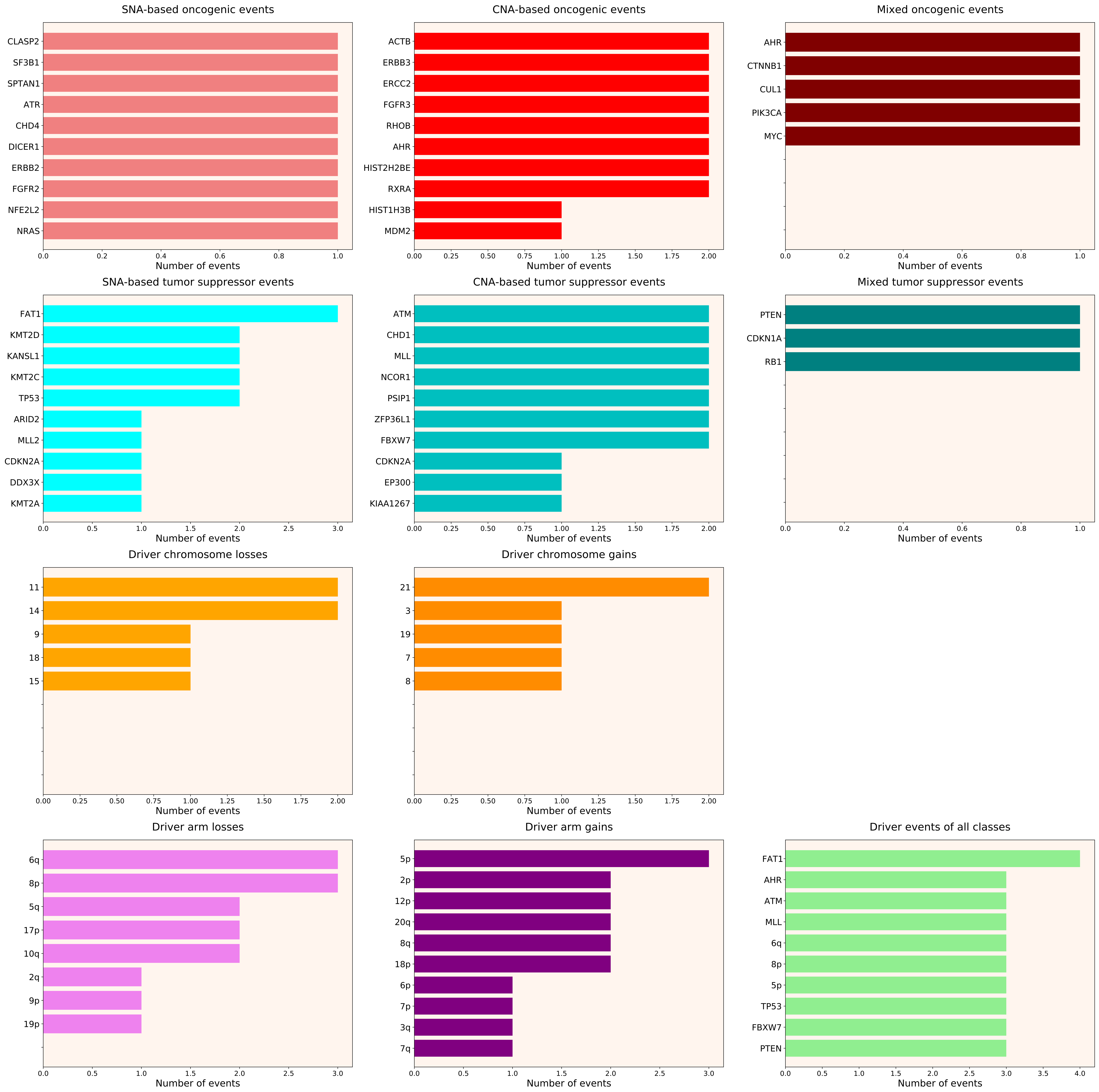

SNA-based oncogenic events
CLASP2
SF3B1
SPTAN1
ATR
CHD4
DICER1
ERBB2
FGFR2
NFE2L2
NRAS
Number of events
CNA-based oncogenic events
ACTB
ERBB3
ERCC2
FGFR3
RHOB
AHR
HIST2H2BE
RXRA
HIST1H3B
MDM2
Number of events
Mixed oncogenic events
AHR
CTNNB1
CUL1
PIK3CA
MYC
Number of events
SNA-based tumor suppressor events
FAT1
KMT2D
KANSL1
KMT2C
TP53
ARID2
MLL2
CDKN2A
DDX3X
KMT2A
Number of events
CNA-based tumor suppressor events
ATM
CHD1
MLL
NCOR1
PSIP1
ZFP36L1
FBXW7
CDKN2A
EP300
KIAA1267
Number of events
Mixed tumor suppressor events
PTEN
CDKN1A
RB1
Number of events
Driver chromosome losses
11
14
9
18
15
Number of events
Driver chromosome gains
21
3
19
7
8
Number of events
Driver arm losses
6q
8p
5q
17p
10q
2q
9p
19p
Number of events
Driver arm gains
5p
2p
12p
20q
8q
18p
6p
7p
3q
7q
Number of events
Driver events of all classes
FAT1
AHR
ATM
MLL
6q
8p
5p
TP53
FBXW7
PTEN
Number of events
